# Hantavirus infections and small mammal diversity in Chile: No differences between protected and unprotected areas highlight the need for public health strategies

**DOI:** 10.1371/journal.pntd.0013668

**Published:** 2025-10-30

**Authors:** Fernando Torres-Pérez, Nicolás Ferrada, Roxana Astudillo, Marcela Ferrés, Pablo A. Vial, Pablo A. Marquet, Alonso Parra, Gregory J. Mertz, R. Eduardo Palma

**Affiliations:** 1 Instituto de Biología, Pontificia Universidad Católica de Valparaíso, Valparaíso, Chile; 2 Departamento de Enfermedades Infecciosas e Inmunología Pediátricas, Pontificia Universidad Católica de Chile, Santiago, Chile; 3 Instituto de Ciencias e Innovación en Medicina, Universidad del Desarrollo, Santiago, Chile; 4 Facultad de Ciencias Biológicas, Pontificia Universidad Católica de Chile, Santiago, Chile; 5 Subsecretaría de Salud Pública, Ministerio de Salud, Santiago, Chile; 6 Department of Internal Medicine, University of New Mexico, Albuquerque, New Mexico, United States of America; 7 Dirección de Postgrado, Universidad de O’Higgins, Rancagua, Chile; Public Health Agency of Canada, CANADA

## Abstract

**Background:**

Zoonotic viruses, such as *Orthohantavirus andesense* (ANDV; the causative agent of hantavirus cardiopulmonary syndrome, HCPS), pose significant public health risks at the human-wildlife interface. Understanding their eco-epidemiological dynamics is critical for elucidating the interplay between reservoir hosts, environmental factors, and spillover to humans. In Chile, the long-tailed pygmy rice rat (*Oligoryzomys longicaudatus*) serves as the primary reservoir for ANDV. This study investigates whether protected areas (PA), which typically support higher biodiversity and stable ecosystems, exhibit lower ANDV seroprevalence compared to unprotected areas (UPA), where anthropogenic disturbances may alter host-pathogen dynamics.

**Methodology:**

Between 2001–2008, we conducted small mammal sampling across 22 sites (11 PA and 11 UPA) in natural landscapes of Chile. Seroprevalence of ANDV was assessed via strip immunoassay, while small mammal diversity was evaluated using standardized trapping protocols and diversity indices. We used similarity percentage analysis to identify species contributing to community dissimilarities and applied Renyi diversity profiles to compare small mammal diversity between area types.

**Main Findings:**

We captured 627 small mammals (PA: 331, 14 species; UPA: 296, 10 species) across 12,898 trap-nights. Seroprevalence in *O. longicaudatus* was identical in PA and UPA (9.5%). No significant differences were found in the relative abundance or seropositivity of *O. longicaudatus* between area types. Ecological indices (Shannon-Wiener, Simpson, richness, evenness) and community composition (ANOSIM) also showed no significant differences. Rényi profiles indicated marginally higher diversity in PA, driven by greater richness and evenness.

**Conclusions:**

These findings suggest that ecological factors, such as habitat type, climatic conditions, and/or human behavior, may play a more critical role in shaping viral prevalence than protection status alone. The study underscores the necessity for consistent public health interventions to mitigate the risk of hantavirus cardiopulmonary syndrome across all environments, particularly in regions where human activities intersect with natural habitats.

## Author summary

This study looked at how a virus that causes a serious disease in humans, spread by wild rodents, behaves in different natural environments in Chile. The study focused on *O. longicaudatus*, the main reservoir of *Orthohantavirus andesense* (ANDV), and compared its presence and infection levels in areas with environmental protection and those without. Over eight years, we studied small mammals in 22 locations across the country. Surprisingly, we found no major difference in the number of infected animals or in the diversity of small mammals between protected and unprotected areas. This challenges the idea that protected areas alone reduce the risk of people catching this virus. The results suggest that other factors—such as the landscape, climate, or human activities—may have a stronger influence on how the virus spreads in nature. This work highlights the importance of monitoring and public health actions in all geographic areas, not just those without protection, to reduce the risk of disease for people who live in or visit natural areas.

## Introduction

Zoonoses, infectious diseases capable of transmission between animals and humans, pose a persistent and dynamic global health threat [1]. These pathogens, frequently harbored within wildlife reservoirs, can cross into human populations via diverse routes, including direct contact, vector-mediated transmission, and inhalation of contaminated aerosols [2]. Among the most pressing concerns are emerging infectious diseases, which can rapidly disseminate across regions and cause severe, often life-threatening outcomes [3]. RNA viruses play a significant role in this landscape, given their high mutation rates, adaptability, and potential for rapid evolution, making them key agents of zoonotic (re-)emergence [4]. Understanding the ecology, transmission, and pathogenesis of these viruses is therefore critical for addressing the challenges posed by zoonoses and emerging infectious diseases [5].

Orthohantaviruses (*Hantaviridae*) comprise a genus of segmented single-stranded RNA viruses, representing a significant global public health concern due to their widespread distribution and the severe clinical outcomes associated with their transmission [6]. In the Americas, these viruses pose a particularly urgent threat, as several genotypes are responsible for hantavirus cardiopulmonary syndrome (HCPS) [7,8], a severe and often fatal disease characterized by acute respiratory distress and cardiovascular collapse [9]. With a fatality rate of approximately 36%, HCPS is recognized as a serious emerging infectious disease, underscoring the need for continued research and public health vigilance [10]. Transmission of orthohantaviruses typically occurs through inhalation of aerosolized rodent excreta or secretions, with rodents serving as the primary reservoir hosts [11,12].

In Chile, the etiologic agent of HCPS is *Orthohantavirus andesense* (ANDV) [13], a unique virus that not only causes severe disease in humans but also is the only known hantavirus where person-to-person transmission has been demonstrated [14,15]. Of note, between 2001 and 2008, 453 cases of HCPS were confirmed in Chile, with a mortality rate of 32.2% (epi.minsal.cl). The primary reservoir of ANDV is the long-tailed pygmy rice rat *Oligoryzomys longicaudatus* (Cricetidae) a species that inhabits heterogeneous landscapes across Chile, spanning from 27°S to 54°S [16–18].

Chile’s National System of Biodiversity and Protected Areas (SBAP) represents a unique ecological and epidemiological context for studying zoonotic diseases such as ANDV. The SBAP encompasses an extensive network of protected areas, including 44 national parks, 24 national reserves, 14 natural monuments, and 45 nature sanctuaries, covering approximately 18.6 million terrestrial hectares (https://datosturismo.sernatur.cl/siet/reporteDinamicoSNASPE). These protected areas (defined as specific, delimited geographic spaces aimed at preserving and conserving the country’s biodiversity, as well as safeguarding natural, cultural heritage, and landscape value, both now and in the long term), attract over 3 million tourists annually, fostering interactions between wildlife and humans near human settlements. Such interactions create opportunities for pathogen spillover, particularly in regions where rodent populations are abundant and where human activities overlap with natural habitats [19].

Rodent abundance, density, and seroprevalence are known to vary across landscapes due to a combination of habitat heterogeneity, climatic oscillations, and species-specific ecological factors [16,20,21]. While complex ecological interactions can either amplify or attenuate viral prevalence [21], protected areas may exhibit distinct eco-epidemiological patterns compared to unprotected areas. For instance, protected areas often harbor elevated rodent populations and species diversity due to reduced human disturbance, which could lead to higher pathogen prevalence [22]. However, increased competition or dilution effects among rodent species may also reduce the likelihood of high viral transmission [23], creating a scenario where protected areas exhibit lower disease prevalence despite higher rodent densities.

Given the ongoing threat of hantavirus transmission in protected areas - which are visited by millions of people each year who may potentially be exposed to ANDV - understanding rodent population patterns, viral prevalence, and their interplay across different habitat types is critical for developing effective public health strategies [10]. Elucidating these eco-epidemiological patterns are essential for protecting human health, particularly in regions where protected areas border human settlements and where tourism and recreational activities increase the likelihood of human-wildlife contact [24,25].

To address these gaps, we conducted an analysis sampling for 8 years to determine the relative abundance and seroprevalence of *O. longicaudatus* and the diversity of small mammals in Chile. By comparing protected areas (PA) with unprotected areas (UPA), we aimed to explore the eco-epidemiological patterns of ANDV infections and their principal reservoir. Our findings provide novel insights into the factors influencing hantavirus transmission in diverse landscapes, offering valuable information for the development of targeted interventions to mitigate the risk of HCPS.

## Materials and methods

### Ethics statement

Permission to trap small mammals was obtained from the Servicio Agrícola y Ganadero (SAG, Chile; permits 17/2000, 7325/2005, 1056/1999), and Corporación Nacional Forestal (CONAF, Chile; permits 10–02/2002, 13–03/2003, 14–99/2004, 24/2004, 07–06/2006). All the National Institute of Health studies were approved by the Institutional Animal Care and Use Committee (IACUC) of the University of New Mexico Health Sciences Centre under protocol number 14–101118-Field-HSC, and the Department of Health and Human Services of the National Institute of Health, Animal Welfare Assurance A5848-01. Small mammals trapping protocols and biosafety procedures for FONDECYT were reviewed and approved by institutional ethics and biosafety review boards at the Pontificia Universidad Católica de Chile (CBB 7/8/2005).

### Sampling

We conducted 22 small mammal samplings across 11 protected and 11 unprotected sites spanning the Coquimbo to Magallanes administrative regions in Chile from 2001 to 2008 (Table 1). To approximate ecological consistency across sites and enhance the robustness of between-group comparisons, we chose sampling areas that fulfilled three criteria to minimize differences in climatic and biogeographic variables based on spatial proximity, temporal synchronicity and ecological similarity: i) the samplings had to be no more than six months apart between PA and UPA, ii) they had to be no more than 100 km apart and iii) they must be included within the same eco-geographic region [16,26].

**Table 1:** Type of area, trapping site, sampling year and *Orthohantavirus andesense* seroprevalence.

Type of area*	Site (Locality, County, Administrative Region)	Sampling Year	Latitude	Longitude	Night traps	Seropositives (SIA)	Total of Micromammals captured	Seroprevalence *(%)*
UPA	Bosque Nagué, Los Vilos, Coquimbo	2003	-31°50’S	-71°31’W	720	4	14	28,6
UPA	Rio Maipo, Santo Domingo, Valparaíso	2002	-33°22’S	-71°22’W	720	0	2	0,0
UPA	Fundo Lisboa, Alhué, Metropolitana	2003	-33°56’S	-71°01’W	570	0	8	0,0
UPA	Tregualemu, Pelluhue, Maule	2008	-35°56’S	-72°44’W	210	0	15	0,0
UPA	Retupel, Cauquenes, Maule	2003	-36°01’S	-72°27’W	300	1	12	8,3
UPA	Vilumanque, Concepción, Biobío	2003	-36°46’S	-73°01’W	218	0	64	0,0
UPA	Lautaro, Lautaro, Araucanía	2002	-38°31’S	-72°25’W	300	0	9	0,0
UPA	Fundo El Condor, Coyhaique, Aysén	2001	-45°33’S	-71°55’W	720	0	16	0,0
UPA	Lago Atravesado, Coyhaique, Aysén	2003	-45°40’S	-72°19’W	360	1	71	1,4
UPA	Porvenir, Porvenir, Magallanes	2008	-53°16’S	-70°09’W	300	1	56	1,8
UPA	Fuerte Bulnes, Punta Arenas, Magallanes	2006	-53°37’S	-70°55’W	600	1	29	3,4
PA	Fray Jorge, Ovalle, Coquimbo 2003	2003	-30°22’S	-71°23’W	720	1	99	1,0
PA	Cerro El Roble, Tiltil, Metropolitana	2003	-32°58’S	-71°01’W	300	0	14	0,0
PA	Quebrada de Córdoba, El Tabo, Valparaíso	2002	-33°26’S	-71°39’W	1080	0	3	0,0
PA	Reserva Nacional Los Ruiles, Chanco, Maule	2003	-35°50’S	-72°30’W	360	2	26	7,7
PA	Reserva Los Queules, Pelluhue, Maule	2008	-35°59’S	-72°41’W	180	1	17	5,9
PA	Parque Nacional Nahuelbuta, Angol, Araucanía	2003	-37°49’S	-73.01W	750	0	16	0,0
PA	Parque Nacional Huerquehue, Pucon, Araucanía	2002	-39°10’S	-71°43’W	990	1	13	7,7
PA	Parque Nacional Queulat, Cisnes, Aysén	2003	-44°27’S	-72°32’W	480	0	25	0,0
PA	Reserva Nacional Rio Simpson, Coyhaique, Aysén 2001	2001	-45°27’S	-72°19’W	500	2	21	9,5
PA	Parque Nacional Pali Aike, San Gregorio, Magallanes	2008	-52°06’S	-69°46’W	1080	0	38	0,0
PA	Reserva Nacional Magallanes, Punta Arenas, Magallanes	2006	-53°07’S	-71°01’W	840	0	59	0,0
TOTAL						15	627	2,4

*UPA: Unprotected area; PA: Protected area

**Fig 1:**
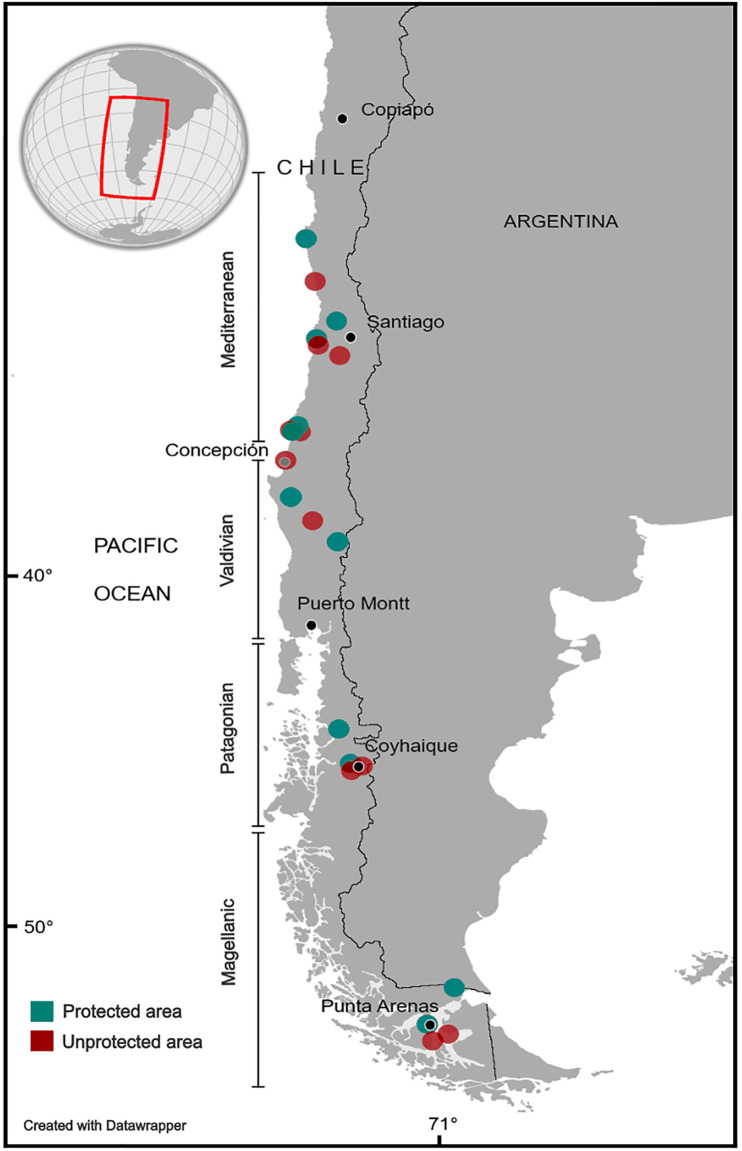
Small mammal trapping sites sampled in protected areas (PA, in green) and unprotected areas (UPA, in red) across Chilean ecoregions. Vertical lines indicate the approximate distribution of major eco-regions (Mediterranean, Valdivian rain forests, Patagonian rain forests, Magellanic subpolar forests). Map created with Datawrapper.

Sampling sites ranged from Fray Jorge National Park, Coquimbo Region (-30º 22’S, -71º 23’W) to Fuerte Bulnes, Magallanes Region (-53º 37’S, -70º 55’W), covering a total of 2,597 kms from north to south (Fig 1). Our sampling scheme encompasses most of the latitudinal distribution of *O. longicaudatus* [27,28]. Small mammals were captured by using live Sherman traps (8 x 9 x 23 cm). The traps were installed with rolled oats and vanilla essence in meadows, thickets, and forests that are part of the habitat of *O. longicaudatus*. The traps were set for 2–6 consecutive nights, varying between 180–1080 traps per sampling site. Captured animals were identified by their external morphology. In the capture and manipulation of small mammals, processing and handling procedures adhered to in accordance with the established protocols [29] and the guidelines set forth by the American Society of Mammalogists [30].

### Hantavirus detection

Captured small mammals were anesthetized, and blood samples were collected via retro-orbital sinus puncture using a heparinized capillary tube. The blood samples were transferred to cryovials, preserved in liquid nitrogen, and subsequently transported to the laboratory for analysis. Additionally, for each specimen, the heart, kidney, spleen, liver, and lung were excised, cryopreserved in liquid nitrogen, and stored for further study. Voucher specimens were fixed in 96% ethanol and deposited in the ‘Colección de Flora y Fauna Professor Patricio Sánchez Reyes’ at Pontificia Universidad Católica de Chile (Santiago, Chile), and the Division of Mammals at the Museum of Southwestern Biology, University of New Mexico (Albuquerque, NM). Antibodies against *Orthohantavirus andesense* were detected in blood samples using a strip immunoassay vacuum-blot test [31]. Briefly, 5 µL of small mammal blood was applied to a 1.6 × 5-cm nitrocellulose membrane with a 0.5-mm × 1.6-mm band containing ~300 ng of affinity-purified recombinant Andes virus N antigen, incubated overnight at room temperature in 1 mL volume. After washing, bound antibodies were detected using alkaline phosphatase−conjugated deer mouse anti-*Peromyscus leucopus* IgG (1:1,000 dilution) and revealed with the BCIP/NBT substrate system (Kirkegaard and Perry Laboratories, Gaithersburg, MD).

### Statistical analysis

For every site we calculated the relative abundance of *O. longicaudatus* ((total of *O. longicaudatus* per site/ total traps nights used in the site) x 100), the trapping effort (total of small mammals captured per site/ total of traps nights used in the site), the relative seropositivity (total of seropositive *O. longicaudatus* per site/ (trapping effort x 100)), and the seroprevalence (positive SIA *O. longicaudatus* per site/ total of *O. longicaudatus* captured per site). To determine the α diversity of small mammals on every site of PA and UPA we calculated the Shannon-Wiener diversity index (H = −Σpi × ln (pi), where pi is the relative proportion of species i in the community), the Simpson diversity index (1 − Σpi2), and the richness (S) and evenness (H/Hmax, where Hmax = ln[S]). We compared the relative abundance and relative seropositivity of *O. longicaudatus* seroprevalence, and diversity indexes using the Wilcoxon-Mann-Withney test between UPA and PA. To determine the β-diversity, we compare small mammal community composition between PA and UPA by performing a one-way analysis of similarity (ANOSIM), which is a permutational non-parametric test based on species abundance [32]; for this analysis we used a Bray-Curtis index as a similarity measure. To represent these results graphically, we applied a non-metric multidimensional scaling (nMDS) approach, which creates a two-dimensional abstract depiction of species composition similarity. The quality of the nMDS representation is assessed through ordination stress, a metric ranging from 0 to 1, where lower values correspond to better fit (stress values ≤ 0.2 are deemed indicative of good ordination). We additionally performed a Similarity Percentage (SIMPER) analysis to assess the extent to which individual species contributed to compositional dissimilarities. Renyi diversity profiles were applied to assess and compare multiple small mammals’ diversity indices between types of areas [33,34]. The comparisons, ANOSIM, SIMPER, Rényi diversity profiles and nMDS were performed on R version 4.4.1 (R Development Core Team, 2021), using the “vegan” package for the diversity indexes and the ANOSIM, SIMPER and Rényi diversity profiles [35].

## Results

**Fig 2:**
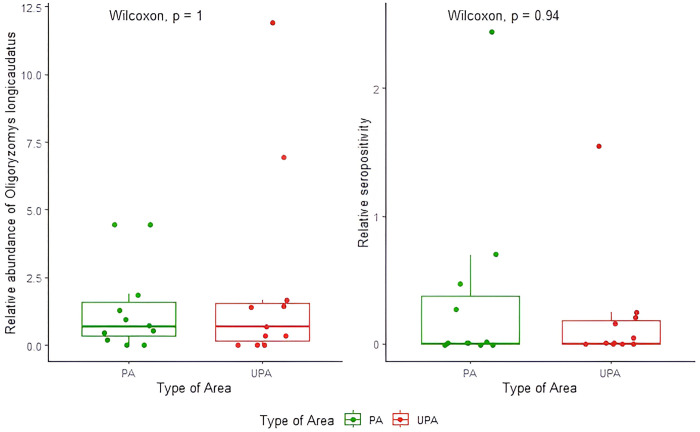
Comparison of relative abundance of *Oligoryzomys longicaudatus* (A) and relative seropositivity (B) between protected areas (PA) in green and unprotected areas (UPA) in red.

We deployed 7,280 traps in PA, capturing 331 small mammal specimens that belonged to 14 species. In UPA, we used 5,618 traps, capturing 296 small mammal specimens of 10 species (S1 Table). Among the 63 specimens of *O. longicaudatus* captured in PA, six resulted positive for ANDV antibodies (seroprevalence of 9.5%). In UPA, 74 specimens of *O. longicaudatus* were captured, with seven seropositives (seroprevalence of 9.5%). Two additional seropositives were detected, one *Abrothrix hirta* in the Reserva Los Queules, Pelluhue (PA) and one *Rattus rattus* in Bosque Nague, Los Vilos (UPA) [36]. The site with the highest seroprevalence (28.6%) in UPA was Bosque Nague, Los Vilos (31°50’S), while in PA, Reserva Nacional Río Simpson, Coyhaique (45°27’S) had a seroprevalence of 9.5%. The comparison of the relative abundance (*p* = 0.947), and relative seropositivity (*p* = 0.658) of *O. longicaudatus*, revealed no significant differences between protected and unprotected areas (Fig 2).

**Fig 3:**
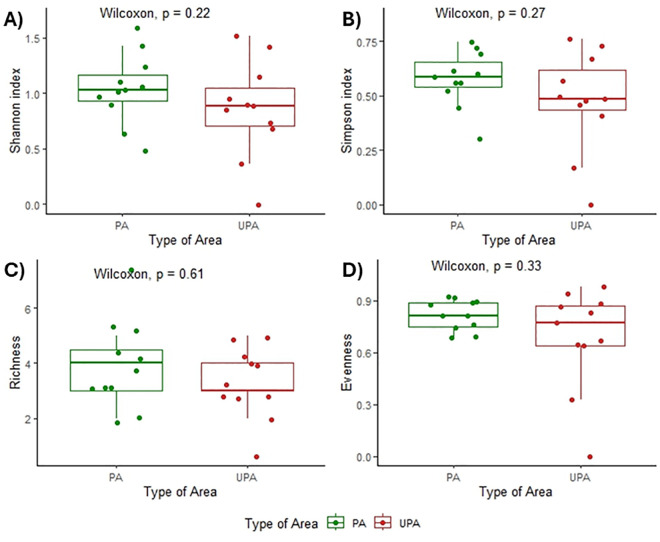
Comparison of overall Shannon-Wiener indexes (A), Simpson indexes (B), Richness (C) and Evenness (D) between protected areas (PA) in green and unprotected areas (UPA) in red.

**Fig 4:**
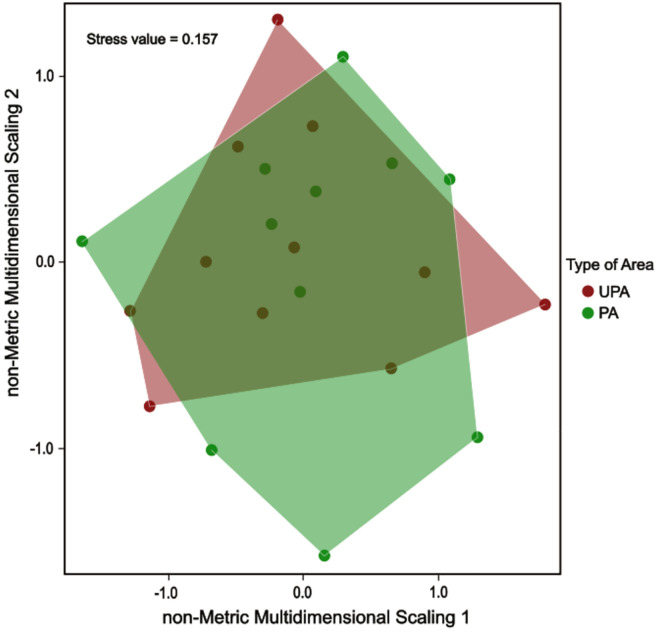
Comparison of the small mammal community’s composition using the analysis of similarities (ANOSIM) between protected areas (PA) and unprotected areas (UPA).

**Fig 5:**
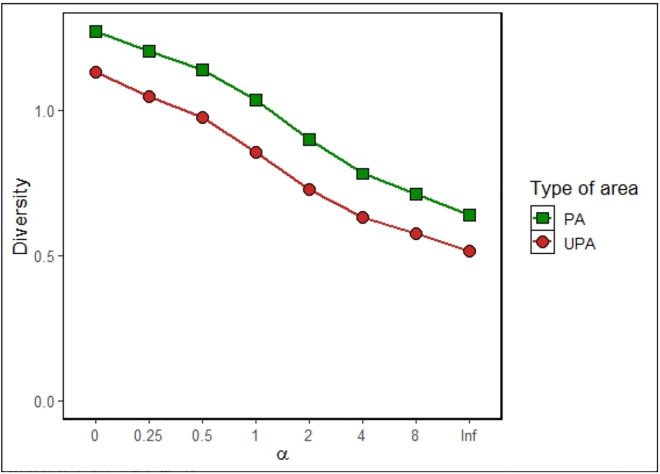
Rényi diversity profiles for Protected Areas (PA) in green and Unprotected Areas (UPA) in red. At lower alpha values, diversity is more influenced by species evenness, whereas higher alpha values emphasize the contribution of dominant species to overall diversity.

Comparative analyses of ecological indices, including the Shannon-Wiener (*p* = 0.212), Simpson diversity (*p* = 0.237), species richness (*p* = 0.611), and evenness (p = 0.293) revealed no significant differences between protected and unprotected areas (Fig 3). No significant differences were detected in micromammal community composition between PA and UPA (ANOSIM R = -0.063, p = 0.886; Fig 4). Similarly, SIMPER analysis consistently failed to identify any species with a statistically significant contribution to the observed compositional dissimilarities (S2 Table). Rényi diversity profiles revealed that PA consistently exhibited a small higher micromammal diversity than UPA across all alpha values, indicating greater species richness (at low α) as well as higher evenness and lower dominance (at high α) in UPA (Fig 5).

## Discussion

The findings of our study reveal unexpected insights into the eco-epidemiological patterns of *Orthohantavirus andesense* (ANDV) in Chile, particularly in relation to its primary reservoir, *Oligoryzomys longicaudatus*. We observed no significant differences in relative seropositivity between *O. longicaudatus* populations in protected areas (PA) and unprotected areas (UPA), with both areas exhibiting a seroprevalence of 9.5%. Similar to higher seroprevalences were reported previously in other protected areas in Argentina [22], whereas seroprevalences were lower in several previous studies in Chile [13,17,37,38], emphasizing the complexity of hantavirus prevalence [39,40]. Our findings emphasize that protection status alone may not be a reliable predictor of ANDV infection. This has important implications for public health interventions, which must remain consistent across both protected and unprotected landscapes to effectively mitigate the risk of human infection, especially in sites that are not seroprevalence hotspots [41].

Although protected areas exhibited marginally higher small mammal diversity than unprotected areas (Rényi), the absence of significant differences across multiple ecological indices - such as the Shannon-Wiener and Simpson diversity measures, species richness, evenness, relative abundance and the analysis of similarity between PA and UPA - further supports that protection status alone may not directly influence ANDV infection rates in reservoir populations. Instead, our findings suggest that other ecological and environmental factors may play a more decisive role in shaping viral prevalence and rodent community structure. For instance, habitat type [16,42,43], climatic conditions [44–49], human behavior [50] have been shown to significantly influence rodent populations and pathogen dynamics. Together with ecological interactions [51–55], these may all play a more decisive role in shaping rodent diversity and viral prevalence [56]. Our findings align with previous studies emphasizing the importance of ecological heterogeneity, rather than protection status, in determining rodent community structure and pathogen transmission [24].

The absence of significant differences in seropositivity and diversity between PA and UPA suggests that viral transmission in *O. longicaudatus* is influenced by a complex interplay of factors that extend beyond species richness alone. Although examples are found for hantavirus infections [57], long term studies may reveal more complex dynamics on how biodiversity impacts mechanisms driving pathogens fluctuations [21,50]. This highlights the importance of integrating both viral and ecological factors into hantavirus surveillance programs [58], as well as the need for a more nuanced understanding of the mechanisms driving ANDV transmission.

Despite the relatively low number of HCPS cases during our sampling period, the high mortality rate (32.2%) highlights the significant threat posed by ANDV in Chile [59], particularly when compared to other RNA viral diseases. For instance, integrating hantavirus prevention into existing tourism infrastructure—such as signage at trailheads, visitor center briefings, and rental equipment hygiene protocols—could reduce exposure risks without requiring costly site-specific interventions. This is particularly concerning in areas where human activities intersect with natural habitats, as these interfaces create opportunities for pathogen spillover [57,58]. Collaborative efforts between public health agencies and park management could further standardize preventive measures, such as discouraging camping near rodent burrows or storing food in rodent-proof containers, across all recreational areas. Protected areas, which often attract significant tourism and recreational activities, represent a unique challenge in this scenario. While our study did not identify significant differences in seroprevalence or diversity between PA and UPA, it highlights the importance of considering broader ecological and anthropogenic factors in the development of targeted public health strategies [39].

This study provides important insights into ANDV seroprevalence and rodent communities across Chile, but certain limitations should be acknowledged. Our sampling for each locality was conducted at a single time point. This cross-sectional design limits our ability to account for temporal variability and infer transmission dynamics, as hantavirus seroprevalence is known to lag behind changes in host population and community structure [21]. Although we applied rigorous geographic and ecological criteria to pair protected and unprotected areas, we did not include fine-scale measurements of environmental disturbance that could offer more detailed context. The study was also not designed to formally test the dilution effect. Lastly, some sites presented no seropositive individuals, which may reduce the statistical power of some comparisons—though these negative detections are themselves informative and contribute to understanding the broader spatial patterns of ANDV prevalence.

## Conclusions

This study contributes to a better understanding of the spatial patterns of ANDV seroprevalence and small mammals’ community composition across Chile. While our results indicate no clear difference in infection prevalence between protected and unprotected areas, they highlight the need to focus prevention efforts on local hotspots of viral activity rather than legal land-use categories alone. The findings also underscore the importance of considering broader ecological and anthropogenic factors when designing hantavirus surveillance and mitigation strategies. Although the study design limits temporal resolution and does not directly assess mechanisms such as the dilution effect, it provides a valuable baseline for future research. Addressing remaining knowledge gaps, such as the role of fine-scale environmental disturbances, climate variability, and rodent host ecology will be key to developing more targeted and effective public health interventions in HCPS endemic regions in Chile.

## Supporting information

S1 Table Type of area, trapping site, year, and relative abundance of small mammals captured in protected and unprotected areas in Chile. (PDF)

S2 Table SIMPER analysis showing the contribution of each species to the average Bray-Curtis dissimilarity in composition between protected (PA) and unprotected areas (UPA). The table includes the average contribution of each species (Average), standard deviation (Sd), contribution-to-variation ratio (Ratio), average abundance in group A (Ava) and group B (Avb), cumulative contribution to dissimilarity (Cumsum), and associated p-value. No species showed a statistically significant contribution to the dissimilarity between groups.
(PDF)
