# Supplementary material for: Hantavirus infections and small mammal diversity in Chile: No differences between protected and unprotected areas highlight the need for public health strategies
Source: PLoS Negl Trop Dis. 2025 Oct 30;19(10):e0013668. doi: 10.1371/journal.pntd.0013668 (PMC12591396; doi:10.1371/journal.pntd.0013668)
Supplement: S1 Table — (PDF) [file pntd.0013668.s001.pdf]

S1 Table. Type of area, trapping site, year, and relative abundance of small mammals captured in protected and unprotected areas in Chile.

Type of area*	Site (Locality, County)	Sampling year	Night traps	*Abrothrix hirta*	*Abrothrix longipilis*	*Abrothrix olivacea*	*Abrothrix sanborni*	*Chelemys megalonyx*	*Dromiciops bozinovici*	*Euneomys chinchilloides*	*Loxodontomys micropus*	*Mus musculus*	*Octodon degus*	*Oligoryzomys longicaudatus*	*Phyllotis darwini*	*Rattus norvegicus*	*Rattus rattus*	*Thylamys elegans*
UPA	Fundo El Condor, Coyhaique	2001	720	1,25	0,00	0,28	0,00	0,00	0,00	0,00	0,69	0,00	0,00	0,00	0,00	0,00	0,00	0,00
UPA	Rio Maipo, Santo Domingo	2002	720	0,00	0,00	0,28	0,00	0,00	0,00	0,00	0,00	0,00	0,00	0,00	0,00	0,00	0,00	0,00
UPA	Lautaro, Lautaro	2002	300	0,00	0,00	2,00	0,00	0,00	0,00	0,00	0,00	0,67	0,00	0,33	0,00	0,00	0,00	0,00
UPA	Bosque Nague, Los Vilos	2003	720	0,00	0,14	0,00	0,00	0,00	0,00	0,00	0,00	0,00	0,00	1,39	0,00	0,14	0,28	0,00
UPA	Fundo Lisboa, Alhué	2003	570	0,00	0,00	0,00	0,00	0,00	0,00	0,00	0,00	0,00	0,00	0,00	0,00	1,05	0,18	0,18
UPA	Lago Atravesado, Coyhaique	2003	360	4,44	0,00	3,89	2,50	0,00	0,00	0,00	1,94	0,00	0,00	6,94	0,00	0,00	0,00	0,00
UPA	Retupel, Cauquenes	2003	300	0,00	0,00	2,33	0,00	0,00	0,00	0,00	0,00	0,00	0,00	1,67	0,00	0,00	0,00	0,00
UPA	Vilumanque, Concepción	2003	218	8,72	0,00	8,26	0,00	0,00	0,00	0,00	0,00	0,00	0,00	11,93	0,00	0,00	0,46	0,00
UPA	Fuerte Bulnes, Punta Arenas	2006	600	3,33	0,00	1,00	0,00	0,00	0,00	0,00	0,17	0,00	0,00	0,33	0,00	0,00	0,00	0,00
UPA	Tregualemu, Pelluhue	2008	210	0,48	0,00	2,38	0,00	0,00	0,00	0,00	0,00	0,00	0,00	1,43	0,00	0,00	2,38	0,48
UPA	Porvenir, Porvenir	2008	900	0,33	0,00	5,67	0,00	0,00	0,00	0,00	0,00	0,00	0,00	0,22	0,00	0,00	0,00	0,00
PA	Reserva Nacional Rio Simpson, Coyhaique	2001	500	1,40	0,00	1,20	0,20	0,00	0,00	0,00	0,00	0,00	0,00	1,40	0,00	0,00	0,00	0,00
PA	Parque Nacional Huerquehue, Pucon	2002	990	0,20	0,00	0,00	0,00	0,00	0,00	0,00	0,61	0,00	0,00	0,51	0,00	0,00	0,00	0,00
PA	Quebrada de Córdoba, El Tabo	2002	1080	0,00	0,00	0,00	0,00	0,00	0,00	0,00	0,00	0,00	0,00	0,19	0,00	0,00	0,00	0,09
PA	Cerro El Roble, Tiltil	2003	300	0,00	0,67	3,00	0,00	0,00	0,00	0,00	0,00	0,00	0,00	0,00	1,00	0,00	0,00	0,00
PA	Fray Jorge, Ovalle, 2003	2003	720	0,00	5,56	2,64	0,00	0,56	0,00	0,00	0,00	0,00	2,22	0,69	1,94	0,00	0,00	0,14
PA	Parque Nacional Nahuelbuta, Angol	2003	750	0,13	0,00	0,40	0,00	0,00	0,00	0,00	0,27	0,00	0,00	0,93	0,00	0,40	0,00	0,00
PA	Parque Nacional Queulat, Cisnes	2003	480	2,71	0,00	0,63	0,00	0,00	0,00	0,00	0,00	0,00	0,00	1,88	0,00	0,00	0,00	0,00
PA	Reserva Nacional Los Ruiles, Chanco	2003	360	1,11	0,00	0,00	0,00	0,00	0,28	0,00	0,00	0,00	0,00	4,44	0,00	1,39	0,00	0,00
PA	Reserva Nacional Magallanes, Punta Arenas	2006	840	1,19	0,00	4,40	0,00	0,00	0,00	0,00	0,83	0,00	0,00	0,48	0,00	0,12	0,00	0,00
PA	P.N. Pali Aike, San Gregorio	2008	1080	0,00	0,00	2,87	0,00	0,00	0,00	0,65	0,00	0,00	0,00	0,00	0,00	0,00	0,00	0,00
PA	Reserva Los Queules, Pelluhue	2008	180	0,56	0,00	3,89	0,00	0,00	0,00	0,00	0,00	0,00	0,00	4,44	0,00	0,00	0,56	0,00

*UPA: Unprotected area; PA: Protected area
