# Supplementary material for: Hantavirus infections and small mammal diversity in Chile: No differences between protected and unprotected areas highlight the need for public health strategies
Source: PLoS Negl Trop Dis. 2025 Oct 30;19(10):e0013668. doi: 10.1371/journal.pntd.0013668 (PMC12591396; doi:10.1371/journal.pntd.0013668)
Supplement: S2 Table — The table includes the average contribution of each species (Average), standard deviation (Sd), contribution-to-variation ratio (Ratio), average abundance in group A (Ava) and group B (Avb), cumulative contribution to dissimilarity (Cumsum), and associated p-value. No species showed a statistically significant contribution to the dissimilarity between groups. (PDF) [file pntd.0013668.s002.pdf]

**S2 Table.** SIMPER analysis showing the contribution of each species to the average Bray-Curtis dissimilarity in composition between protected (PA) and unprotected areas (UPA). The table includes the average contribution of each species (Average), standard deviation (Sd), contribution-to-variation ratio (Ratio), average abundance in group A (Ava) and group B (Avb), cumulative contribution to dissimilarity (Cumsum), and associated p-value. No species showed a statistically significant contribution to the dissimilarity between groups.

| Species | Average | Sd | Ratio | Ava | Avb | Cumsum | p-value |
|---|---|---|---|---|---|---|---|
| *Abrothrix olivacea* | 0.2207 | 0.2009 | 1.0984 | 10.4500 | 10.0900 | 0.3070 | 0.797 |
| *Oligoryzomys longicaudatus* | 0.1547 | 0.1256 | 1.2316 | 5.7300 | 6.7300 | 0.5220 | 0.211 |
| *Abrothrix hirta* | 0.1293 | 0.1310 | 0.9868 | 3.4500 | 6.1800 | 0.7020 | 0.624 |
| *Loxodontomys micropus* | 0.0440 | 0.0717 | 0.6136 | 1.3600 | 1.1800 | 0.7630 | 0.553 |
| *Abrothrix longipilis* | 0.0376 | 0.0956 | 0.3927 | 3.8200 | 0.0900 | 0.8150 | 0.562 |
| *Rattus norvegicus* | 0.0363 | 0.0759 | 0.4774 | 0.8200 | 0.6400 | 0.8650 | 0.719 |
| *Rattus rattus* | 0.0231 | 0.0455 | 0.5067 | 0.0900 | 0.8200 | 0.8970 | 0.539 |
| *Phyllotis darwini* | 0.0192 | 0.0437 | 0.4400 | 1.5500 | 0.0000 | 0.9240 | 0.215 |
| *Octodon degus* | 0.0119 | 0.0384 | 0.3104 | 1.4500 | 0.0000 | 0.9410 | 0.586 |
| *Euneomys chinchilloides* | 0.0109 | 0.0364 | 0.3009 | 0.6400 | 0.0000 | 0.9560 | 0.378 |
| *Abrothrix sanborni* | 0.0106 | 0.0278 | 0.3836 | 0.0900 | 0.8200 | 0.9710 | 0.775 |
| *Thylamys elegans* | 0.0096 | 0.0237 | 0.4050 | 0.1800 | 0.1800 | 0.9840 | 0.480 |

| | | | | | | | |
|---|---|---|---|---|---|---|---|
| *Mus musculus* | 0.0064 | 0.0233 | 0.2747 | 0.0000 | 0.1800 | 0.9930 | 0.674 |
| *Chelemys megalonyx* | 0.0030 | 0.0096 | 0.3104 | 0.3600 | 0.0000 | 0.9970 | 0.586 |
| *Dromiciops bocinovici* | 0.0020 | 0.0069 | 0.2951 | 0.0900 | 0.0000 | 1.0000 | 0.344 |
